# The Role of Oxidative Stress in Sarcoidosis

**DOI:** 10.3390/ijms222111712

**Published:** 2021-10-28

**Authors:** Sara Solveig Fois, Sara Canu, Alessandro Giuseppe Fois

**Affiliations:** 1 Department of Medical, Surgical and Experimental Sciences, University of Sassari, Viale San Pietro 43, 07100 Sassari, Italy; agfois@uniss.it; 2 Respiratory Diseases Operative Unit, University Hospital of Sassari, 07100 Sassari, Italy; sara.canu@aousassari.it

**Keywords:** sarcoidosis, oxidative stress, antioxidant, biomarker

## Abstract

Sarcoidosis is a rare, systemic inflammatory disease whose diagnosis and management can pose a challenge for clinicians and specialists. Scientific knowledge on the molecular pathways that drive its development is still lacking, with no standardized therapies available and insufficient strategies to predict patient outcome. In recent years, oxidative stress has been highlighted as an important factor in the pathogenesis of sarcoidosis, involving several enzymes and molecules in the mechanism of the disease. This review presents current data on the role of oxidative stress in sarcoidosis and its interaction with inflammation, as well as the application of antioxidative therapy in the disease.

## 1. Introduction

Sarcoidosis is a systemic inflammatory disorder of unknown cause whose pathogenesis is mainly attributed to T-lymphocytes, mononuclear phagocytes, and the accumulation of non-caseating granulomas. Exposure to an unidentified foreign antigen, aberrant innate immune response and possibly a genetic predisposition are all suspected factors leading the pathobiology. There are variable disease manifestations, but the lungs are affected in over 90 percent of cases with pulmonary involvement accounting for most of the morbidity and mortality. Disease course is characterized by episodic flare-ups and remissions that are impossible to predict. Prognosis is extremely variable, and up to one third of patients may develop unremitting disease resulting in progressive organ impairment [1].

Despite the recent advances in understanding the mechanisms underlying this disease, sarcoidosis remains a challenging diagnosis, and there are still no validated biomarkers that can assist the clinician in diagnosis or prediction of disease outcome [2,3,4]. Approved therapies are lacking, with corticosteroids being still the first drug of choice to improve symptoms and prevent organ damage and limited evidence regarding the usefulness of other immunosuppressants.

Defined as an imbalance between the production and the elimination of oxidant compounds, oxidative stress may underlie some of the molecular mechanisms that characterize sarcoidosis pathogenesis. The role of oxidative stress has been extensively documented in several lung diseases, including chronic obstructive pulmonary disease (COPD) and lung fibrosis [5,6]. Even though the study of oxidative stress in sarcoidosis has been poorer than in other diseases over the past few decades, the presence of redox imbalance has been documented in sarcoidosis too.

This review evaluates oxidative stress in sarcoidosis and discusses the potential role of antioxidants and possible targets to reduce oxidative stress in sarcoidosis patients. 

## 2. Oxidative Stress and the Lung

In living systems, oxidative stress is linked to the presence of reactive chemical species that are naturally produced at a cellular level. Reactive oxygen species (ROS) and reactive nitrogen species (RNS) are the main protagonists of oxidant production.

ROS is a term that collectively indicates chemical species such as superoxide radical (O_2_^−^), hydrogen peroxide (H_2_O_2_), hydroxyl radical (OH) and singlet oxygen (^1^O_2_), among others. They represent a type of oxygen-derived intermediates that are partially reduced, highly reactive and unstable [7]. ROS are normally generated as by-products of aerobic metabolism of oxygen in the mitochondria, but there are other extramitochondrial sources such as NADPH (NOXs) oxidation enzymatic reactions that take place in the phagosomes [8]. ROS are intrinsic to cellular functioning as homeostatic modulators and cell signaling factors. They are also a key component of the immune response: for instance, it has been shown that ROS induce antiviral activity through interferon-gamma regulation in nasal epithelial cells in response to influenza infection [9]. However, ROS can have harmful effects because of their ability to oxidize and modify cellular structures (proteins, lipids, nucleic acids). Oxidization of macromolecules leads to cell dysfunction and ultimately cell death and tissue damage. To counterbalance the inevitable accumulation (the so-called “burden”) of ROS and their detrimental effects, cells deploy an inherent antioxidant defense system to protect themselves from ROS-induced damage. This system is based mainly on the activity of antioxidant enzymes such as superoxide dismutase (SOD), catalase (CAT), and glutathione peroxidase (GPx), all of which work synergically to maintain oxidant species at adequate levels and minimize tissue damage.

RNS are a family of molecules that are produced starting with the rapid reaction of nitric oxide (NO) with superoxide to form peroxynitrite (ONOO^−^). Peroxynitrite can cause nitrosative damage to various biological targets, but it can also react with other molecules to form additional types of RNS, including nitrogen dioxide (NO_2_) and dinitrogen trioxide (N_2_O_3_). Increased RNS production has been shown in acute lung injury and adult respiratory distress syndrome (ARDS), suggesting a role in the pathophysiology of inflammatory lung diseases [10]. Moreover, increased production of the potent oxidant peroxynitrite has been observed in the lungs of patients with idiopathic pulmonary fibrosis (IPF) [11].

The delicate balance between the never-ending production and removal of ROS/RNS may be disturbed under certain conditions, such as exposure to an excess supply of oxygen (hyperoxia), inflammation, or when antioxidant defenses are impaired or limited. [12,13]. Under these circumstances, the antioxidant capacity becomes saturated, and the burden of oxidant species is uncontrolled. This phenomenon is generally described as oxidative stress (OS) and results in cellular and tissue damage (e.g., lipid peroxidation). There is undisputed evidence that OS plays a role in the pathogenesis of numerous diseases such as cardiovascular disease, cancer, diabetes, neurodegenerative diseases, post-ischemic perfusion injury, aging, and other chronic and degenerative conditions in humans [4]. In the last years, researchers have focused their attention on the elucidation of the exact role of OS in disease onset and development, and particularly on finding new approaches for the treatment of chronic diseases that are based on the idea that decreasing oxidative stress could prevent or cure them [14].

**Figure 1:**
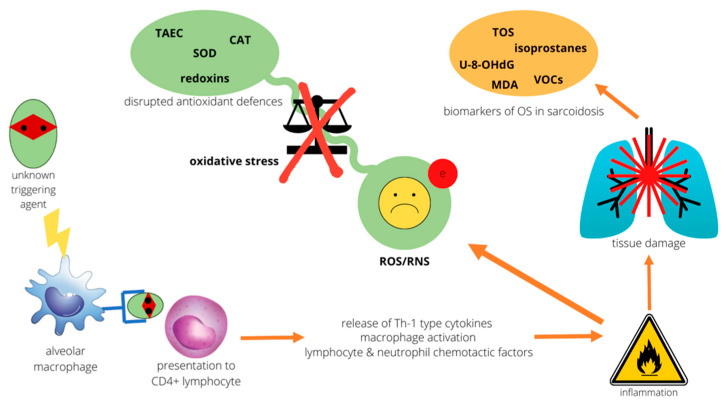
Overview of the role of oxidative stress in sarcoidosis.

Of all human organs, the lung is particularly susceptible to damage caused by OS. For a start, the respiratory surface area is exposed to higher oxygen tension than other tissues. The direct injury to cells caused by air oxygen is exemplified by the notorious detrimental effects of hyperoxia that can be observed when an increased fractional inspired oxygen (FiO_2_) is administered clinically [15]. The lung surface is also a prime filter for inhaled pollutants, cigarette smoke, irritants, and ultrafine particulates. The literature regarding their oxidative damage on the pulmonary epithelium is extensive [16,17,18]. Finally, the lung is vulnerable to bacterial, fungal, or viral infections, to which the resident immune cells respond by deploying oxidizing defenses via the NADPH pathway in macrophages and granulocytes. This results in a large production of ROS/RNS, which is further amplified by the host inflammatory response (see Figure 1) [8,19]. Increased oxidation levels have a potential role in the onset and development of various lung conditions and have been shown to play a part in the pathobiology of airway disease, ARDS, lung cancer, and idiopathic pulmonary fibrosis [20,21,22,23].

## 3. Evidence of Redox Imbalance in Sarcoidosis

The immunopathogenesis of sarcoidosis entails a complex interplay of immune cells, mediators, and cytokines. The formation of sarcoid granulomas is thought to be initiated by the presentation of a foreign trigger by antigen-presenting cells (macrophages or dendritic cells) to CD4+ T lymphocytes. In turn, T lymphocytes amplify the immune response through release of Th1-type cytokines such as interleukins (IL) 12, 18 and 27, interferon gamma (IFN-γ), and tumor necrosis factor alpha (TNF-α) at sites of inflammation. These mediators promote macrophage accumulation, activation, and aggregation, while others such as IL-6 and IL-8, which are produced by macrophages, act as lymphocyte and neutrophil chemotactic factors, respectively [24]. These events result in the development of granulomatous inflammation, although the exact details of this process are still not clear [25,26].

In sarcoidosis, the activation of the macrophage capability to produce toxic oxygen molecules might play a crucial role in developing the tissue damage. In this regard, the first pieces of evidence regarding increased oxidative burden in sarcoidosis date back to over 30 years ago. Pioneering studies on lung alveolar cell populations demonstrated that resident macrophages from sarcoid patients with a high-intensity lymphocytic alveolitis produce higher amounts of superoxide anion, both spontaneously and in the presence of a triggering agent. This capability is dependent on IFN-γ levels, and macrophages can alternate states of high or low activity depending on local environment [27,28,29]. While Lenz et al. first observed oxidatively modified proteins in the bronchoalveolar lavage fluid of patients with sarcoidosis in 1996, it was not until the late nineties that the first studies on single components as markers of OS in sarcoidosis were conducted with the specific aim to quantify oxidative burden and to identify biomarkers that could correlate with disease activity, as listed in Table 1 [30].

In the study of lung diseases, oxidative stress biomarkers can be evaluated in several biological matrices. In COPD, for example, evidence of OS has been gathered from markers in the peripheral blood and urine, as well as from bronchoalveolar lavage fluid (BALF), induced sputum and exhaled breath condensate (EBC) [31]. In sarcoidosis, the study of OS in peripheral blood is even more warranted by the systemic nature of the disease. In fact, as we mentioned before, sarcoidosis can involve virtually any other organ, suggesting that the pathobiology of the disease is not necessarily tied to the cellular population of the lung only. In this regard, systemic redox imbalance in the context of sarcoidosis was demonstrated by Koutsokera et al. [32]. In their study, blood samples from 35 patients with disease stability, and 13 healthy volunteers were evaluated with a spectrophotometric method that measures the derivatives of reactive oxygen metabolites, known as the D-ROM test. Results showed that oxidative stress levels were significantly higher in patients with sarcoidosis compared to those of normal subjects, but oxidative stress levels in those patients receiving corticosteroids (18 out of 35) did not differ significantly compared to controls. Interestingly, this study provided evidence of a sustained oxidative burden even during a stable phase of the disease, indicated by clinical, functional, and radiological criteria. This finding may imply that the underlying pathophysiological events that occur during sarcoidosis persist even when the disease is not active, and that OS plays a significant role in this context. An increase in total oxidant status (TOS) in sarcoid patients was confirmed further in later studies by Ivaniševic et al. with Erel’s method, an assay that is based on the oxidation of ferrous ion to ferric ion in the presence of various oxidant species. In their studies, Ivaniševic et al. focused their research on the alterations in the circulating lipid profile in large cohorts of patients with sarcoidosis [33,34].

### 3.1. Biomarkers of Lipid Peroxidation: MDA, Isoprostanes, and VOCs

In vivo measurement of products of lipid peroxidation such as malondialdehyde (MDA), exhaled volatile alkanes, and prostaglandin-like compounds has received much attention over the last decades. Lipid peroxidation is, in fact, a major effect of oxidative damage, and there is abundant evidence showing the association between the levels of these biomarkers and the development of various diseases [35].

Malondialdehyde (MDA) is a product of radical-induced peroxidation of fatty acids and is one of the most studied biomarkers of oxidative stress in several diseases, usually with thiobarbituric acid (TBA) assay methods (TBARS) [36,37]. In a study by Uzun et al. on the levels of antioxidant defense in patients with active sarcoidosis, serum TBARS-MDA concentrations were found to be significantly increased in sarcoidosis. Furthermore, serum MDA levels were associated with disease activity and negatively correlated with the levels of antioxidant defense. Consistently with the fact that it is the final product of lipid peroxidation, MDA positively correlated with the increase in oxidized LDL in this study [38]. Unsurprisingly, MDA-modified LDLs have already been proposed as an independent biomarker for atherosclerosis [39]. In sarcoidosis however, the reliability of MDA assays is questionable due to the intrinsic instability of MDA in biological fluids [40]. Moreover, the quantification of MDA with the TBARS method may be affected by the ability of thiobarbituric acid to react with several other components other than MDA, which can overestimate the presence of the marker [36]. For these reasons, concerns regarding the reliability of MDA as a biomarker for OS have been raised.

Isoprostanes have emerged as reliable markers of oxidative damage in a wide variety of diseases and are one of the most used approaches to assess free radical injury in patients with lung disease. It has been postulated that isoprostanes may have a potential role as mediators of oxidant injury, a theory that has obvious therapeutic implications [41,42]. 8-epi-prostaglandin F2 alpha (8-isoprostane) is a prostaglandin-like compound formed in vivo through the free radical-catalyzed peroxidation of arachidonic acid [43]. Elevated 8-isoprostane concentrations in blood, urine, BALF or EBC have been reported in smokers and several lung diseases associated with redox imbalance such as COPD, asthma, ARDS and IPF [44,45,46,47,48,49,50]. Increased levels of 8-isoprostane have been detected in the BAL fluid of sarcoidosis subjects as early as 1998, as shown in a study conducted by Montuschi et al. The aim of their research was to measure 8-isoprostane in BAL in normal subjects and to compare them to those observed in patients with pulmonary fibrosis (9 subjects with IPF, 8 subjects with SS-ILD) and sarcoidosis (10 patients). Results showed that 8-isoprostane was increased in the BALF of patients with sarcoidosis, although the highest levels of free radical activity as reflected by 8-isoprostane concentrations were found in the groups of patients with IPF and SS-ILD. In the sarcoidosis group, the authors observed a negative trend between 8-isoprostane concentrations and lymphocyte count and a positive trend with macrophage count. No correlation between 8-isoprostane and lung function tests was observed in any group of patients [45]. Fifteen years later, Malli et al. explored differences in 8-isoprostane levels in serum and in BAL in 16 IPF and 55 sarcoidosis patients, as well as in 17 healthy subjects. The authors demonstrated increased serum levels of 8-isoprostane in all patient groups versus controls, with concentrations particularly elevated in sarcoidosis patients who also presented significantly increased 8-isoprostane BAL levels when compared to IPF patients. This study confirmed the implication of redox imbalance in both diseases [51].

The presence of 8-isoprostane in EBC has also been investigated with great interest, as this procedure has the advantage of being non-invasive and thus may represent a suitable candidate for routine disease follow-up. However, there are conflicting studies regarding the ability of 8-isoprostane in EBC to correlate with disease activity in sarcoidosis. In their study published in 2004, Psathakis et al. measured increased concentrations of this marker of oxidative stress in the EBC of 30 patients with sarcoidosis compared to 12 control subjects. Furthermore, 8-isoprostane levels correlated with disease activity as measured by serum angiotensin-converting enzyme (ACE) level, serum calcium level, and pulmonary function test results. 8-Isoprostane levels in patients with non-active disease did not differ from those in healthy subjects [52]. A later study from Piotrowski et al. confirmed elevated levels of 8-isoprostane in EBC in sarcoidosis patients, but in this case, the measurement of eicosanoids as activity markers was discouraged as no correlation was found between 8-isoprostane and BALF lymphocyte percentage [53]. For this work, the authors enrolled 28 sarcoidosis patients and 17 healthy subjects with the aim to verify the relationship between EBC and BALF eicosanoids, and the percentage, number, and activity of cells in BALF. 8-isoprostane and leukotrienes were measured in EBC by enzyme immunoassay. In BALF, eicosanoids, cell count, percentage, and superoxide production were estimated. Results showed that although both eicosanoids and leukotrienes were elevated in EBC in sarcoidosis patients, there was a lack of correlation with BALF lymphocyte percentage that did not encourage the measurement of eicosanoids as activity markers. In fact, prior to this study, the origin of eicosanoids in the lung had not been precisely defined, and inflammatory cells present in the airway lumen had only been presumed to be the main source. On the other hand, the authors found a positive correlation of EBC 8-isoprostane and BALF leukotrienes concentrations with the percentage of eosinophils in BALF, and a higher percentage of eosinophils in BALF from patients with more advanced stages of sarcoidosis, thus suggesting that the measurement of eicosanoids in EBC could have a prognostic value.

Volatile organic compounds (VOCs) such as ethane and pentanes are products of the interaction of ROS with biological components and can be measured in the exhaled breath [54]. Despite being a relatively recent field of research, this type of breath analysis has already shown promising potential in the non-invasive detection and monitoring of oxidative stress in several lung diseases including COPD, asthma, and lung cancer [55,56,57]. In a study on interstitial lung diseases that included 6 patients with sarcoidosis, elevation of exhaled ethane was observed during disease exacerbations on gas samples obtained at hospital admission and seemed to normalize with clinical improvement after 3 weeks. Ethane concentration, which was analyzed by gas chromatography, was at lower levels in steroid-treated and stable patients. This was also the case for other ILDs such as IPF. This study suggests not only that lipid peroxidation occurs in these patients, but also that exhaled ethane could be an indicator of inflammatory response, as well as a predictor of disease activity in sarcoidosis and other ILDs [58].

### 3.2. Oxidatively Damaged DNA

Oxidative damage to the DNA has been studied in sarcoidosis, although to a lesser extent than other OS biomarkers and specifically in cardiac sarcoidosis. 8-hydroxydeoxyguanosine (8-OHdG), an oxidized nucleoside, is the most frequently studied DNA alteration, and is considered a marker that can be measured in urine and serum for assessing total oxidative burden [59]. Urinary levels of this biomarker have recently been associated with disease activity in cardiac sarcoidosis. In fact, a study conducted by Kobayashi et al. in 2015 measured urinary 8-OHdG levels in 31 patients with cardiac sarcoidosis, 28 patients with dilated cardiomyopathy (DCM), and 30 control subjects. Results showed that U-8-OHdG levels in sarcoidosis patients were higher than those in controls, and significantly higher in a subgroup of patients with active disease (based on 18F-fluorodeoxyglucose positron emission tomography) than in non-active sarcoidosis and DCM patients. In active sarcoidosis patients, U-8-OHdG levels were re-examined at 6 months after corticosteroid treatment to assess response and were found to be significantly decreased in proportion with the decrease in the focal cardiac uptake of 18F-FDG. Thus, the authors demonstrated that U-8-OHdG is a potentially clinically useful biomarker for evaluating inflammatory activity of cardiac sarcoidosis and monitoring the effectiveness of corticosteroid therapy. A year later, the same research group proved that 8-OHdG can also be considered a powerful predictor of ventricular arrhythmias and cardiovascular-related death in sarcoidosis [60,61]. Despite the small sample size of the study, these findings are encouraging and represent an important milestone in the ongoing effort to find additional markers of disease activity in sarcoid patients with cardiac involvement.

### 3.3. Disrupted Mitochondrial Homeostasis

In the mitochondria, ROS are normally generated as by-products of aerobic metabolism of oxygen during electron transport chain activity. Inappropriate ROS production resulting from mitochondrial impairment is thought to underlie various chronic lung diseases, potentially contributing to lung cell dysfunction and disease progression [62,63].

The role of mitochondrial dysfunction in sarcoidosis has only just started to be unveiled. It was observed that mitochondria do show notable morphological changes in the capillary endothelial cells in patients with sarcoidosis, as demonstrated in the study carried out by Mochizuki et al., who in 2011 examined tissues of the bronchus and lung obtained from 16 patients and 11 controls and observed a close relationship between mitochondria and lipid droplets in capillary endothelial cells of the respiratory tract. The authors postulated that this relation may be involved in the pathogenesis of sarcoidosis [64]. The genetics of mitochondrial dysfunction in sarcoidosis have also been tentatively investigated. In a recent characterization of the mitochondrial genome in 40 patients with IPF and 85 patients with sarcoidosis, sequence analysis identified a total of 53 mutations with a mutation frequency of 95% among IPF patients and 81% among patients with sarcoidosis. In sarcoidosis, 25/45 mutations were exclusively expressed in the disease, while 17/45 defects were observed in healthy controls as well, even though their occurrence remained predominant in sarcoidosis. Of the 45 mutations identified, the authors revealed 4 novel defects in flanking genes. In IPF, 15/32 mutations only occurred in the disease, while 12/32 were only predominant in the disease group compared to healthy controls. Despite these results, none of the detected mitochondrial mutations have been shown to correlate directly with lung diseases pathogenesis, and the mechanisms of how these single mutations could translate into a deregulation of oxidative chain reactions remain unknown. However, the finding that novel mutation combinations were solely expressed in disease indicates that a mitochondrial-mediated pathogenic pathway may underlie both IPF and sarcoidosis [65]. Further proof that dysmetabolism in the mitochondria is responsible for the altered redox balance in sarcoidosis come from an elegant proteomic study conducted on BALF [66]. In this pilot study, Bhargava et al. characterized the proteins in BAL cells from 4 controls and 4 sarcoidosis cases matched for age, sex, race, and smoking status. Results showed that 272 out of 4306 cellular proteins were differentially expressed in sarcoidosis BAL cells compared to controls. These proteins mapped to novel pathways such as integrin-linked kinase and IL-8 signaling and previously implicated pathways in sarcoidosis, including phagosome maturation, clathrin-mediated endocytic signaling and redox balance. Several enzymes that mapped to NRF2-mediated oxidative stress response were also higher in sarcoidosis compared to controls. NRF2 is a transcription factor that is a central mitochondrial regulator of nearly all cellular antioxidant proteins in the lungs and other organs [67]. BAL fluid proteins were also found to be differentially expressed and mapped to aryl hydrocarbon signaling, communication between innate and adaptive immune response, integrin, PTEN and phospholipase c signaling, serotonin and tryptophan metabolism, autophagy, and B cell receptor signaling. Additional pathways that were different between progressive and non-progressive sarcoidosis in the BALF included CD28 signaling and PFKFB4 signaling. This study demonstrated the power of contemporary proteomics to reveal novel molecular mechanisms in sarcoidosis, with applications that may be helpful to identify biomarkers for diagnosis and prognosis and novel molecular therapeutic targets.

**Table 1:** Overview of the most important biomarkers of oxidative stress in sarcoidosis.

Biomarker	Matrice	Advantages	Disadvantages	Reference
total oxidative status	blood	can be determined with various methods easy to reproduce correlates with disease activity	high oxidative status in blood may not be illustrative for that of matrices that are relevant to disease	[32,33,34]
MDA	blood	easy to determine correlates with disease activity	sampling can affect results	[38]
8-isoprostane	blood BALF EBC	can be determined in various matrices correlates with disease activity, advanced stages, and pulmonary function tests	no correlation with BALF lymphocyte count in one study	[51,52,53]
exhaled ethane	EBC	determined with noninvasive method correlates with disease activity	single study on sarcoidosis	[58]
U-8-OHdG	urine	correlates with disease activity	single study on sarcoidosis	[60]

## 4. Diminished Antioxidant Defenses

The lung has an efficient built-in antioxidant defense system that operates through an enzymatic cascade that prevents the production of oxidant species. At the same time, scavengers convert oxidants to fewer toxic compounds, blocking the secondary production of intermediate species. The lung epithelial cells are rich with constitutively acting antioxidant enzymes such as glutathione (GSH) peroxidase, catalase (CAT), redoxins, and superoxide dismutase (SOD) that maintain the redox balance within the cells. In the airway secretions, albumin and mucin also show spontaneous antioxidant activity through their exposed sulfhydryl groups. Furthermore, the epithelial lining fluid contains several other non-enzymatic antioxidants such as ascorbic acid (vitamin C), alpha-tocopherol (vitamin E) and uric acid.

Diminished antioxidants defenses have been demonstrated in sarcoidosis, both locally and systemically. The most studied antioxidants in sarcoidosis are listed in Table 2. An approximate evaluation of the oxidant/antioxidant balance in the blood of patients with sarcoidosis can be reached by assessing total antioxidant capacity (TAC), an analyte that is a measure of the number of free radicals scavenged by a test solution. With this method, Boots et al. were the first to demonstrate a significant decrease in the total plasma antioxidant capacity as well as in the blood levels of important endogenous antioxidants such as glutathione, vitamin C and uric acid in sarcoidosis, while exploring at the same time the potential of the flavonoid quercetin to mitigate inflammation. [68]. In this work, TAC was measured by relating the free radical scavenging properties of the blood to that of the synthetic antioxidant Trolox, i.e., the Trolox equivalent antioxidant capacity (TEAC value). The study compared 20 patients to 11 healthy controls matched for age, gender, and dietary behavior, indicating that the observed low antioxidant levels were not the result of a different dietary intake, but could be attributed to the elevated production of ROS present in sarcoidosis. Furthermore, results showed that the severe cases of sarcoidosis displayed a trend towards a lower total plasma antioxidant capacity as well as a lower GSH level, which the authors suggested as evidence that the severity of the disease might be related to the level of oxidative stress.

### 4.1. Redoxins

Redoxins are small redox enzymes with H_2_O_2_ scavenging capacity that have been described as key modulators of oxidative stress response pathways through their ability to rapidly regulate the activation of transcription factors involved in the antioxidant defense system [69]. The research on their potential impact and function in human health and pathological conditions has increased exponentially over the past few decades, with important milestones reached for the main domains of thioredoxins (Trxs), glutaredoxins (Grxs) and peroxiredoxins, which have been studied in many OS-related diseases, including sarcoidosis [70]. In the lung, both Trxs and Grxs have an essential role in the protection against exogenous oxidants and are widely expressed in bronchial cells and alveolar macrophages, a location that emphasizes their importance in the primary defense system of the lung [71].

The involvement of redoxins in pulmonary sarcoidosis has been investigated in the early 2000s with the help of immunohistochemistry. In the year 2000, Koura et al. first associated thioredoxins with sarcoidosis [72]. Their analysis was conducted on 20 patients with clinically active disease and histological evidence of non-caseating granulomas from diagnostic lung biopsy specimens taken by transbronchial, thoracoscopic, or open biopsies. Immunostaining with anti-Trx antibody of granulomas in the lung and lymph node tissue showed strong reactivity, as opposed to no significant concentration in lung tissue specimens from 12 control subjects. Positive staining was more prominent in macrophages, epithelioid cells, and Langhans-type giant cells of the granulomas, but not in lymphocytes, which conversely showed positive staining for interleukin-2 receptor (IL-2R) antibodies. Based on these results, the authors postulated that since Trx has been shown to enhance the proliferative response of lymphocytes to IL-2 and IL-2R inducing activity increased Trx concentrations may contribute to lymphocyte proliferation at the sites of disease activity in patients with sarcoidosis [73]. In 2003, another study was conducted on lung biopsies by Tiitto et al., which reported moderate to intense expression of the Trx system in both alveolar macrophages and pulmonary granulomas of 18 sarcoidosis patients. However, the presence of weak or moderate Trx activity was also detected in the airway epithelium and in alveolar macrophages of 6 healthy controls as well as in individuals with different histological diagnoses, specifically usual interstitial pneumonia (UIP) and desquamative interstitial pneumonia (DIP). This study suggested that a Trx-rich antioxidant profile in alveolar macrophages is of major importance in the resistance of human lung to oxidants, but also highlighted that the expression of Trx is associated with regenerative and inflammatory processes occurring in interstitial lung diseases including sarcoidosis and may thus serve as a marker of cell regeneration and inflammation [74]. By contrast, the expression of Grxs seem to be reduced in sarcoidosis, as depicted by the only study available on the subject which investigated the expression of Grx in human healthy lung versus interstitial lung diseases including sarcoidosis (17 cases), but also extrinsic allergic alveolitis (EAA, also known as hypersensitivity pneumonitis or HP) and UIP (5 cases) [75]. In this work by Peltoniemi et al., the expression of Grx, which functions as a glutathione-dependent hydrogen donor, was found to be upregulated in the alveolar macrophages of healthy lung, a finding that is consistent with the well-established notion that alveolar macrophages are oxidant-resistant cells with a primary role in lung protection. The impairment of this antioxidant system seems to occur both in sarcoidosis and in other OS-related lung diseases and was here documented by a much lower expression of Grx in alveolar macrophages of sarcoidosis and allergic alveolitis compared with controls, while bronchial epithelium of these diseases revealed no Grx immunoreactivity at all. The authors assessed the possible mechanisms underlying Grx expression by exposing cultured airway epithelial cells to TNF-α and transforming growth factor beta (TGF-β), which are major cytokines in the pathogenesis of inflammatory and fibrotic lung disorders. Overproduction of TGF-β is associated with functional impairment in patients with pulmonary sarcoidosis, while TNF-α has a prominent role in the inflammatory process seen in the disease [76]. The authors found that Grx expression, similarly to glutathione itself, is downregulated by TGF-β in vitro, while TNF-α exposure caused no clear effect [77].

### 4.2. CAT and SOD

CAT is the oldest known and first discovered antioxidant enzyme. It is expressed in every organ, particularly in tissues containing relatively high activity such as liver, kidney, and red blood cells [78]. In addition to its dominant catalytic activity (decomposition of H_2_O_2_ into water and oxygen), CAT can also catalyze the oxidation of various metabolites and toxins, including formaldehyde and alcohols [79]. In the lung, CAT is mainly found in macrophages, pneumocytes and fibroblasts and acts as an endogenous antioxidant enzyme. SOD, with its cofactors zinc, copper, and manganese is a scavenging antioxidant enzyme that converts the superoxide radical O_2_^−^ to H_2_O_2_ and oxygen. H_2_O_2_ is then reduced to water by CAT and GPx. SOD also inhibits the oxidative inactivation of nitric oxide (NO). In studies conducted by Lakari et al. on granulomatous and interstitial lung diseases, manganese-SOD (MnSOD) and CAT levels were compared to control subjects. In a work published in the year 1998, the group examined lung biopsies of granulomatous diseases: 22 patients with pulmonary sarcoidosis and 10 with extrinsic allergic alveolitis [80]. Intense MnSOD immunoreactivity was observed in most granulomas of both diseases compared with control subjects, especially in Langhans-type giant cells and to a lesser degree in epithelioid cells. BAL fluid staining also revealed significantly higher amounts of MnSOD immunoreactivity in the macrophages of sarcoidosis and EAA than in controls. In contrast, CuSOD was not induced in either of these diseases. These findings are in accordance with studies suggesting than MnSOD appears to be low in healthy lung, and it is induced during cytokine-mediated protection [81,82,83]. However, MnSOD immunoreactivity in this work was not specifically characteristic for sarcoidosis since granulomas of both pathological conditions showed intense positive staining. In a later study, the same research group investigated the expression of both MnSOD and CAT in biopsies of sarcoidosis and EEA as well as in different types of parenchymal lung diseases such as UIP and DIP. Results showed elevated MnSOD expression in all diseases compared with healthy subjects, especially in type II pneumocytes, alveolar macrophages and granulomas. In contrast to MnSOD, CAT was constitutively and abundantly expressed in normal lung, but was not upregulated in any of the lung diseases [80]. A more recent study by Ivanišević et al. measured blood SOD activities while investigating oxidative stress status and antioxidant defense parameters in 213 treated sarcoidosis patients and 90 controls [34]. The study included 94 patients with acute sarcoidosis and 114 with chronic sarcoidosis, with pulmonary or extrapulmonary disease. All patients were being treated with corticosteroids (prednisolone) and/or immunosuppressants (methotrexate). Serum SOD activity was measured with an epinephrine assay and was found to be lower in patients compared to healthy subjects. Serum total antioxidant status (TAS) was also decreased, while OS parameters such as serum and erythrocyte total oxidative status (TOS), serum pro-oxidant-antioxidant balance (PAB) and MDA were significantly higher.

### 4.3. GSH and PON

Glutathione is a low-molecular-weight antioxidant that is essential to balance oxidative stress in most living systems and the most abundant thiol in animal cells. It exists in reduced (GSH) and oxidized (GSSG) states. In the reduced state, the thiol group is a source of one reducing equivalent and glutathione disulfide (GSSG) is thereby generated, with the GSSG/2GSH ratio serving as a good indicator of the cellular redox state [84]. GSH protects cells by reducing reactive oxygen species and deactivating free radicals, but it is also an important signaling molecule and participates in several metabolic and regulatory processes, including protection from inflammation and immunity. In the lung, the GSH system is one of the major first-line antioxidant defenses and a regulator for innate immunity [85]. It is also a powerful inhibitor of the inflammatory response via the regulation of the transcription factors activator protein-1 (AP-1) and nuclear factor-kB (NF-κβ), which are redox-sensitive. Various lung diseases have been associated with glutathione deficiency, including HP, IPF and ARDS [86,87,88,89,90]. At the same time, a protective role of exogenous GSH (or its synthetic precursor NAC) against inflammatory pathologies of the lung has been demonstrated in multiple animal models [85]. In sarcoidosis, the role of GSH is yet to be fully investigated. In 2002, a comparative study by Rothkrantz-Kos and colleagues found decreased erythrocyte NADPH a co-factor required to maintain adequate levels of reduced glutathione, in a considerable percentage of female patients with pulmonary sarcoidosis [91]. Furthermore, a significant decrease in the blood levels of GSH in sarcoidosis patients, compared to healthy controls, has been demonstrated by Boots et al. in their previously mentioned study on the antioxidant status associated with inflammation in sarcoidosis and the potential role for antioxidants [68].

Serum paraoxonase-1 (PON1) is a glycoprotein which binds to circulating high-density lipoprotein (HDL) particles and preserves their function through inactivation of lipid peroxidation. It was also shown to inhibit low-density lipoprotein (LDL) oxidation, thus protecting endothelial cells from apoptosis [92]. Thanks to these antioxidant, anti-apoptotic and anti-inflammatory actions, PON1 is thought to influence the risk of developing cardiovascular disease and has been named a “cardioprotective enzyme” [93,94,95,96]. In the lung, PON1 has been detected through immunohistochemical evidence in type 1 pneumocytes, but its physiological role in the context of lung antioxidant activity has not yet been described [97]. However, there are a few publications on the role of PON1 activity in patients with asthma and COPD [98,99,100] and on its impact on lung cancer behavior and progression by antioxidative function and controlled ROS accumulation [101].

In sarcoidosis, downregulation of PON1 has been observed by Uzun et al. in 2008. Their work focused on blood analysis of patients with active (26) and inactive (37) sarcoidosis plus 48 healthy subjects. Serum PON1 activity, MDA levels and oxidized LDL were determined. Results showed that PON1 levels were significantly lower in the active disease state group than both the inactive form and control groups, with a negative correlation with MDA levels. Oxidized LDL levels were significantly increased in both the active and inactive groups than the control group, a result suggesting that the increasing oxidative stress in sarcoidosis might be due to increased lipid peroxidation [38]. In this sense, PON1 levels could hypothetically be regarded as a potential biomarker for sarcoid disease activity.

**Table 2:** Overview of the most relevant antioxidants in sarcoidosis.

Antioxidant	Matrice	Characteristics	Comments	Reference
TAC	blood	easy to reproduce correlates with disease activity	low antioxidant status in blood may not reflect status of matrices that are relevant to disease	[68]
redoxins	lung biopsy specimens	Trx-rich antioxidant profile in sarcoidosis Grx-poor antioxidant profile in sarcoidosis in single study, as observed in other OS-related diseases	Trx may serve as marker of cell regeneration/inflammation	[72,74,75]
CAT	lung biopsy specimens	abundant in normal lung, no altered expression seen in sarcoidosis		[80]
SOD	EBC	low in healthy subjects, MnSOD upregulated in sarcoidosis	downregulated in patients treated with steroids/immunosuppressants	[34,80]

## 5. Antioxidant Interventions in Sarcoidosis

The relevance of redox imbalance to the pathogenesis of sarcoidosis, coupled with the correlation of many biomarkers with disease activity, have understandably induced the scientific community into evaluating oxidative stress as a potential novel source of therapeutic targets. Current therapies for sarcoidosis focus primarily on pharmacologically limiting inflammation, and include corticosteroids, immunosuppressants and anti-TNF-α agents, which are all associated with variable response and important side effects. Oxidative stress provides a set of pathways in the pathogenesis of sarcoidosis that are yet to be fully explored and that are intertwined with inflammation. The high production of oxidant species that occurs in sarcoidosis consumes antioxidants. For this reason, it can be hypothesized that antioxidant supplementation might be beneficial in sarcoidosis treatment, but there have been very few studies on the subject.

N-acetylcysteine (NAC), a thiol-containing mucolytic agent and the precursor of glutathione, has a well-established antioxidant activity [102]. Consequently, increasing NAC levels has already been tested as a potential therapeutic goal in various OS-related lung diseases such as COPD and IPF. Although ex vivo studies on the effect of NAC on alveolar macrophages from IPF patients showed downregulation of LPS-induced TNF-α production, a very recent study comparing NAC and placebo in IPF showed no impact on the rate of decline in pulmonary function tests or rate of death [103,104]. A recent clinical trial of NAC in COPD patients was also unable to show any clinical benefit [105]. With regards to sarcoidosis, the effect of NAC therapy was examined in a recent study protocol conducted by Hamzeh et al. on a small group of 14 patients. In this double blinded study, 11 subjects were randomized to active therapy and 3 to placebo for 8 weeks, and bronchoscopy with bronchoalveolar lavage was performed pre- and post-therapy. While the primary endpoint was to assess TNF-α production from LPS-stimulated and unstimulated BAL cells, measures of oxidative stress (GSH and 8-OHdG) levels in the BAL were secondary outcomes. Results showed that NAC failed to suppress ex vivo TNF-α production from BAL cells despite effects in vitro, a discrepancy that the authors attributed to the poor pulmonary bioavailability of oral NAC in the lung tissue. Conversely, there was a 59% increase in BAL GSH levels after NAC treatment, an encouraging result that can still support the idea that antioxidant therapy may have a role in the management of sarcoidosis, although no clinical trials have yet followed up these findings [106].

In recent years, antioxidant research worldwide has particularly focused on quercetin. Quercetin is a natural compound of the flavonoid group of polyphenols. It is abundant in many fruits, vegetables, seeds, and grains, and is also used as a dietary supplement [107]. Quercetin has displayed strong antioxidant properties and is, in fact, the most active scavenger of ROS and RNS both in vitro and in vivo [108]. It is also known for exerting anti-inflammatory activity and significantly inhibiting ex vivo LPS-induced production of TNF-α via modulation of the NF-κβ system [109,110]. This dual effect has been confirmed in sarcoidosis in an interesting interventional study by Boots et al, which followed up their previous investigations on the potential role for antioxidants in the disease. In the present work, 12 participants were randomized to a supplementation of 4 × 500 mg quercetin administered orally within 24 h, and 6 to placebo. Results showed that one day quercetin supplementation improved the antioxidant defense, indicated by the increased total plasma antioxidant capacity. Moreover, quercetin supplementation also reduced markers of oxidative stress (indicated by plasma MDA levels) and inflammation (measured as plasma ratios of TNF-α /IL-10 and IL-8/IL-10 as pro-inflammatory markers) in the blood of sarcoidosis patients, with the effects appearing to be more pronounced when the levels of the oxidative stress and inflammation markers were higher at baseline. Blood GSH levels were unaffected by supplementation in all patients [111]. This study highlights the relevance of quercetin as a potential therapeutic agent with a unique ability to reduce oxidative stress as well as inflammation, a quality that warrants testing for long-term use in sarcoidosis and, most of all, investigating the effect of lung function. Indeed, targeting TNF-α in sarcoidosis is not a new approach: Infliximab, a readily available anti-TNF-α medication, has been studied in patients with glucocorticoid-refractory disease, with encouraging results. The issue warrants future clinical investigations [112,113].

Another drug that has been shown to have antioxidant activity is Pirfenidone, a medication used for the treatment of IPF that works primarily through downregulation of the production of growth factors and procollagens I and II, although the exact mechanisms are elusive. Pirfenidone shows superoxide radical scavenging activity beyond its well-known antifibrotic effect and has shown a potent antioxidant role in experimental cirrhosis [114]. Currently, a phase IV clinical study on Pirfenidone for progressive fibrotic sarcoidosis is ongoing.

## 6. Conclusions

Sarcoidosis is an inflammatory condition of unknown cause that usually occurs in the lungs and lymph nodes, but it can affect almost any organ. Some individuals develop disabling disease despite corticosteroids and immunosuppressants, with an impelling need for effective tools to predict disease progression and drug efficacy and for better therapeutic approaches to preserve organ function. Over recent years, persuasive evidence has accumulated demonstrating that oxidative stress is a critical component of the pathology and progression of sarcoidosis and is intertwined with pathways of inflammation that are implicated in the disease (see Figure 1). Increased levels of renowned markers of oxidative stress such as oxidatively damaged macromolecules and products of lipid peroxidation in various biological matrices in sarcoidosis patients has stimulated great interest in searching for simple and non-invasive ways to predict disease outcome, but consensus on their relationship with disease activity has not yet been reached. The measurement of eicosanoids in exhaled breath condensate, for instance, positively associated with clinical indicators of diseases activity in one study but failed to correlate with BALF lymphocyte percentage in another work. Disrupted antioxidant defenses seem to associate with disease development, as it has been documented for major enzymatic systems such as glutathione, superoxide dismutase, and paraoxonase-1. However, solid clinical trials on the use of supplement antioxidants in sarcoidosis have so far been missing, despite encouraging results coming from single centers investigations, for example, on the use of quercetin, which has interestingly shown dual antioxidant and anti-inflammatory effects in sarcoidosis. Disrupted mitochondrial homeostasis, resulting in increased production or reactive species has also been documented, with proteomic studies holding much potential into exploring novel molecular avenues in development and progression of sarcoidosis, but the results need to be evaluated in further large-scale structural and functional studies to move from correlation to causality.

## Data Availability

Not applicable.
